# Intervertebral Disc Elastography to Relate Shear Modulus and Relaxometry in Compression and Bending

**DOI:** 10.3390/bioengineering13040437

**Published:** 2026-04-08

**Authors:** Zachary R. Davis, P. Cameron Gossett, Robert L. Wilson, Woong Kim, Yue Mei, Kent D. Butz, Nancy C. Emery, Eric A. Nauman, Stéphane Avril, Corey P. Neu, Deva D. Chan

**Affiliations:** 1 Weldon School of Biomedical Engineering, Purdue University, West Lafayette, IN 47907-2032, USA; 2 Paul M. Rady Department of Mechanical Engineering, University of Colorado, Boulder, CO 80309-0427, USA; 3 Department of Engineering Mechanics, Dalian University of Technology, Dalian 116024, China; 4 School of Mechanical Engineering, Purdue University, West Lafayette, IN 47907-2035, USA; 5 Department of Ecology and Evolutionary Biology, University of Colorado, Boulder, CO 80309-0334, USA; 6 Department of Biomedical Engineering, University of Cincinnati, Cincinnati, OH 45221-0048, USA; 7 Mines Saint-Étienne, Université Jean Monnet, INSERM, U 1059 Sainbiose, 42023 Saint-Étienne, France; 8 Biomedical Engineering Program, University of Colorado, Boulder, CO 80309-0521, USA; 9 BioFrontiers Institute, University of Colorado, Boulder, CO 80303, USA; 10 Department of Radiology and Imaging Sciences, Indiana University School of Medicine, Indianapolis, IN 46202, USA

**Keywords:** quantitative MRI (qMRI), relaxometry, elastography, displacement-encoded imaging, intervertebral disc

## Abstract

Intervertebral disc degeneration is the most recognized cause of low back pain, characterized by the decline in tissue structure and mechanics. Image-based mechanical parameters (e.g., strain, stiffness) may provide an ideal assessment of disc function that is lost with degeneration, but unfortunately, these remain underdeveloped. Moreover, it is unknown whether strain or stiffness of the disc may be predicted by MRI relaxometry (e.g., *T*_1_ or *T*_2_), an increasingly accepted quantitative measure of disc structure. In this study, we quantified *T*_1_ and *T*_2_ relaxation times and compared to in-plane strains measured with displacement-encoded MRI within human cadaveric discs under physiological levels of compression and bending. Using a novel inverse approach, we then estimated shear modulus in orthogonal image planes and regionally compared these values to relaxation times and 2D strains. Intratissue strain depended on the loading mode, and shear modulus in the nucleus pulposus was typically an order of magnitude lower than the annulus fibrosus. Relative shear moduli estimated from strain data derived under compression generally did not correspond with those from bending experiments. Only one anatomical region showed a significant correlation between relative shear modulus and relaxometry (*T*_1_ vs. µ_rel_, coronal plane under bending). Together, these results suggest that future inverse analyses may be improved by incorporating multiple loading conditions into the same model and that image-based elastography and relaxometry should be viewed as complementary measures of disc structure and function to assess degeneration in future studies.

## 1. Introduction

Low back pain is the leading cause of chronic disability in industrialized Western societies [1]. Although the causes for low back pain are likely multifactorial, lumbar intervertebral disc degeneration is widely recognized as the most prevalent factor [2]. However, the link between degeneration and pain remains controversial because structural indications of degeneration—typically assessed via radiography, computed tomography, and magnetic resonance imaging (MRI)—are also found in asymptomatic individuals [3]. Efforts to resolve this discrepancy are hindered in part by the inherent subjectivity and qualitative nature of these clinical assessments. Furthermore, clinical imaging approaches lack sensitivity to tissue-level changes to the composition and mechanical behavior of a degenerated disc. Therefore, the noninvasive quantification of early disc degeneration remains a significant challenge.

Disc degeneration, even its earliest form, is characterized by a breakdown of the extracellular matrix (ECM) and a loss of water and proteoglycan content [4]. Because quantitative MRI (qMRI) can relate changes in relaxation time (e.g., *T*_1_, *T*_2_) with alterations to the water, proteoglycan, and collagen content [5,6], relaxometry biomarkers can be used to evaluate the extent of disc degeneration, as well as other musculoskeletal tissues and conditions, as well reviewed by others [7,8,9,10]. *T*_1_ and *T*_2_ have been correlated to the degeneration grade [11,12] and tissue macromolecule composition, including proteoglycan [13] and collagen [14]. Furthermore, relaxation times indicate a spatial distribution of biochemical content, enabling the localization of tissue degeneration [15]. However, because the relaxation time also depends on factors such as collagen orientation [16], age [12], and mechanical loading history [17], the interpretation of relaxometry is complex and may not directly correlate to the mechanical behavior of a disc under load. Assessments of mechanical function, from benchtop mechanical testing [18] to noninvasive measurement of intratissue strains [19], may provide independent and complimentary imaging biomarkers for the evaluation of disc degeneration.

Image-based biomechanics approaches, including MRI-based strain mapping, have emerged as powerful tools for quantifying intervertebral disc mechanics under load. MRI has been used for full-field strain measurement of the disc during mechanical loading, enabling the spatially resolved assessment of tissue deformation. MRI-based approaches to calculate tissue deformation and strain in a disc include warp field image registration [20], digital image correlation [21,22,23,24], and displacements measured under applied loading by MRI (dualMRI) [25,26]. dualMRI uses a combination of displacement encoding with stimulated echoes (DENSE) synchronized with a loading device to acquire phase data that are directly proportional to tissue displacement. dualMRI has been used previously to characterize strain behavior in intervertebral discs and can be readily adapted for the measurement of in vivo tissue deformations [25,26].

Full-field displacements and strains, such as those derived from dualMRI, further enable elastography—the estimation of material property maps (e.g., shear modulus) using inverse modeling. Ex vivo and in vivo MRI elastography (MRE) have produced multi-dimensional shear modulus maps of discs [27,28,29,30]; this method is generally limited to high frequency (~1000 Hz) shear waves that may not reflect the properties of the viscoelastic disc under normal (low frequency; ~1 Hz) activities like walking or bending. In contrast, dualMRI synchronizes mechanical loading at more physiologically relevant loading frequencies (i.e., 1–2 orders of magnitude lower than MRE) to cyclic phase-contrast image acquisition.

Whereas the tissue content and material properties dictate but are not altered by loading, the mechanical behavior of the disc is inextricably linked to the applied loading and other boundary conditions. Inverse modeling can be used to estimate mechanical parameters using image-based full-field strain [31]. These inverse modeling approaches have been successful in estimating stress tensors [32], the stiffness ratio [33], and shear moduli [33]. Recent developments have improved the accuracy of calculating the shear modulus of soft tissue-like materials [34]. Therefore, our objectives in this study were twofold: (1) to implement and evaluate the performance of inverse modeling for the estimation of relative shear moduli from dualMRI of human cadaveric discs and (2) to investigate the correlation of mechanical parameters (strain, relative shear modulus) with MRI-based biomarkers associated with tissue composition (*T*_1_ and *T*_2_) as functions of the region within the disc.

## 2. Materials and Methods

### 2.1. Specimen Preparation

Human lumbar L4–L5 motion segments from three healthy donors (1 female and 2 male, 35 ± 13 yrs, range: 22–48 yrs, height: 172 ± 12 cm, weight: 92 ± 17 kg, Pfirrmann grade: 2.3 ± 0.2) were procured (Upstate New York Transplant Services (UNYTS), Inc., Buffalo, NY, USA). Excess tissues, including the pedicles, laminae, superior and inferior articular processes, and the transverse and spinous processes, were removed while preserving the anterior and posterior longitudinal ligaments and the intervertebral disc. The samples were visually inspected during dissection to check for features indicative of disc degeneration (i.e., concise margins between the vertebral bodies and the discs, ample disc space, lack of osteophytes).

**Figure 1:**
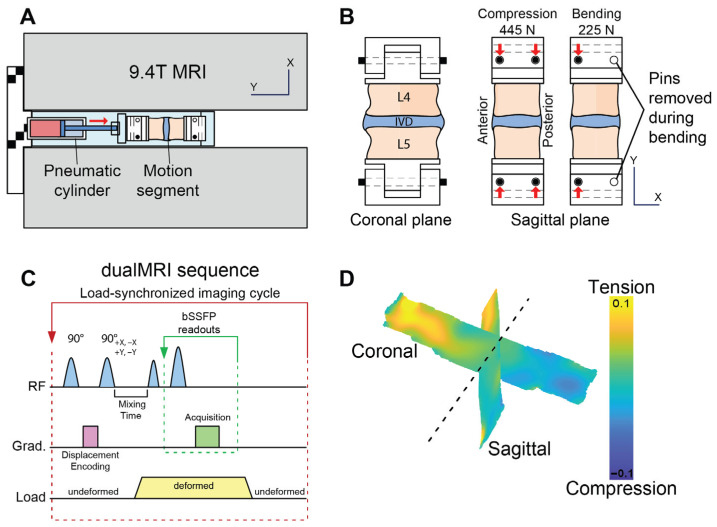
Experimental setup for MRI-based elastography measurements throughout the interior of the intervertebral disc in compression and bending. (**A**) MRI-compatible electro-pneumatic loading system designed for a 9.4-Tesla MRI system. An *x*-*y* coordinate system is defined to enable orientation of the images. (**B**) Support pin configuration permits toggle of loading modes from centric to eccentric axial compression, the latter of which generates bending loads in the intervertebral disc (IVD) between the fourth lumbar (L4) and fifth lumbar (L5) vertebrae. (**C**) Loading profile synchronized with displacement encoding with stimulated echoes for displacements measured under applied loading by MRI (dualMRI). (**D**) Example smoothed displacement maps measured in orthogonal imaging planes.

L4 and L5 vertebral bodies were secured using fiberglass resin into a sample holder, which was connected to an electro-pneumatic loading system compatible with a 9.4-Tesla horizontal bore MRI system (Bruker GMBH, Ettlingen, Germany; Figure 1A) [35]. The design of the sample rig allowed for a quick interchange of loading modes from axial compression to bending mode by the removal of a support pin (Figure 1B). To prevent desiccation, the tissues were wrapped with PBS-soaked gauze throughout the cyclic loading and MR imaging experiment, and PBS was replenished as needed.

### 2.2. T_1_ and T_2_ Mapping

Prior to biomechanical loading, *T*_1_ and *T*_2_ mapping of the disc was performed in both the sagittal and coronal planes, approximately through centroid of the disc. The scan parameters were field of view = 64 × 64 mm^2^, spatial resolution = 250 × 250 µm^2^, and slice thickness = 2 mm. For *T*_1_ relaxation time mapping, a fast spin echo acquisition was used with multiple repetition times (TR = 100, 300, 500, 1000, 2000, 4000 ms) and an echo time (TE) of 10 ms. For *T*_2_ relaxation time mapping, the fast spin echo acquisition parameters were TE = 20, 60, 100, 141, 181, 221, 261, 301 ms and TR = 4000 ms. Image analysis software (Paravision 5.1, Bruker GMBH, Ettlington, Germany) was used to estimate *T*_1_ and *T*_2_ at each pixel of interest with exponential fitting. Only pixels with R^2^ > 0.7 were considered for analysis.

### 2.3. dualMRI and Strain Mapping

Using dualMRI, 2D Green–Lagrange strains (*E_xx_*, *E_yy_*, *E_xy_*) were measured within coronal and sagittal imaging planes under cyclic compression and bending [35]. In the axial compression mode, 445 N was applied along the superior–inferior axis to simulate the force experienced during normal gait [36,37]. In the bending mode, the posterior support pins were removed to create a 1.33 cm offset to the applied compression (225 N, Figure 1B). This created a 3.0 N∙m bending moment in the anterior direction, which is a typical magnitude within the lumbar spine under non-strenuous movements [37,38]. Loading was held for 2 s to permit MRI imaging and followed by a 3 s unloaded recovery (Figure 1C). Preconditioning cycles were applied for 30 min to achieve a steady-state response and minimize motion artifacts before the start of load-synchronized imaging. Given these loading conditions, we chose two orthogonal planes (sagittal and coronal) that would contain the largest expected in-plane displacements under axial loading and bending. Since axial compression occurred in the in anterior-to-posterior direction, the largest displacements within the disc are expected at the anterior endplate, which would be captured by both the sagittal and coronal planes. For bending, the largest displacements would be expected in the plane of the bending moment.

Following the methods for dualMRI at 9.4 T [35], displacements were encoded at 0.32 rad/mm, and phase cycling was used to eliminate artifacts [39]. The acquisition of images, using the same resolution and field of view as *T*_1_ and *T*_2_ mapping, was accomplished with balanced steady-state free precession (bSSFP, TE/TR = 1.607 ms/3.215 ms, flip angle = 25°). Custom scripts in MATLAB (R2012a, MathWorks, Natick, MA, USA) were used to calculate displacements, which were directly proportional to the phase difference between reference and displacement-encoded scans. Displacements were smoothed using 10 iterations of a 5 × 5 Gaussian kernel applied within the whole disc region of interest. Strains were estimated with respect to the imaging coordinate system (*E_xx_*, *E_yy_*, *E_xy_*), and the in-plane principal strains (*E*_1_*, E*_2_) and maximum shear strain (*γ_max_*) were also determined.

### 2.4. Inverse Modeling

We estimated the relative shear modulus ($\mu_{rel}$) of each disc utilizing an iterative inverse approach, minimizing the gap between the measured and computed displacement fields throughout the region of interest in L2 norm [40], such that the lowest tissue modulus has a value of 1, with stiffer tissues having $\mu_{rel}>1$. We solved the inverse problem using the adjoint-based inverse method and utilized in-house code [41]. Although this inverse approach has been evaluated in phantoms [42], it has not been applied to deformations measured nondestructively from MRI. Briefly, the computed displacement field was obtained by solving the forward problem using the finite element method. In the forward model, we assumed that the disc satisfies an incompressible isotropic linear elastic constitutive behavior, an assumption based on the high water content of disc tissues. In the inverse problem, displacement boundary conditions were prescribed on the top and bottom edges of the disc, based on the dualMRI data. In the incompressible and linear elastic model, only the shear modulus needs to be determined (Equation (1)).

(1) σ=2με+pI 

The stress tensor σ can be expressed in terms of the strain tensor ε, shear modulus μ, identity tensor I, and hydrostatic pressure p for an incompressible linear elastic material. Since the tissue displacement was measured only within single 2-mm thick image planes through the disc, we also assumed that the disc was in a 2D plane strain state. In the finite element forward simulation, we use stabilized finite elements to address volumetric locking related to incompressibility [42]. In the inverse modeling, we introduced a regularization term in the cost function to smooth the reconstructed elastic property distribution and avoid overfitting. The optimization problem was solved by the quasi-Newton method where the derivative of the objective function with respect to every shear modulus in the domain of interest is required [42]. To reduce the computation cost, an adjoint-based method was employed to solve the inverse problem. In this framework, the shear modulus was defined at the nodal level and treated as the optimization variable. At each minimization iteration, the nodal shear moduli were updated using the L-BFGS algorithm. This iterative process terminated when the difference of the objective function values or the associated gradients between two neighboring iterations were lower than a threshold (10^−16^). No assumptions of material homogeneity were made, and material properties were allowed to vary spatially. These procedures [42] were used to estimate the relative shear modulus from the image-based strains that resulted from cyclic compression and bending. Since only the displacement condition was used in the model, the shear modulus can only be mapped to a multiplicative factor [42]. The relative shear modulus was calculated within a manually segmented region of interest representing each disc, independently for each loading condition and image plane.

### 2.5. Monte Carlo Simulations

To establish the error and identify any bias of the inverse approach, we generated a smoothed displacement map based on experimental data to establish a ground truth data set and used this to perform Monte Carlo simulations of the estimation of the relative shear modulus using MATLAB. Added random noise was based on the previously measured precision of dualMRI derived displacements (mean = 0 µm, standard deviation = 20 µm) performed on a high-field MRI system [43], and we applied the ground truth displacement map to generate 100 different noisy displacement maps that were then smoothed. The relative shear modulus was estimated as described above for the ground truth displacement map and the 100 displacement map simulations. The root mean squared error (RMSE) was calculated at every pixel in the region of interest, using the ground truth displacement map as the reference; precision was calculated as a pooled standard deviation.

### 2.6. Image and Statistical Analysis

Displacement maps from dualMRI were used to register *T*_1_ and *T*_2_, which were measured in the undeformed disc, onto the deformed disc geometry, the image space within which strains were calculated and moduli estimated. Relaxation times (*T*_1_, *T*_2_) and mechanical parameters (*µ*, 2D strains) were evaluated for all discs using MATLAB. Data are reported as the mean ± standard deviation across the entire region of interest. Paired *t*-tests were used to compare the average relative shear moduli estimated from compression or bending. Distributions of these parameters were also visualized for each disc using histograms for qualitative comparison.

To investigate the spatial correlations and relationships among different relaxation time and mechanical parameters, regional analyses were performed. Correlations between the relative shear modulus as estimated from compression (*µ_rel_*_(*c*)_) and the relative shear modulus as estimated from bending experiments (*µ_rel_*_(*b*)_) were calculated. Correlations between relaxation-time maps (*T*_1_, *T*_2_) and relative shear moduli (*µ_rel_*_(*c*)_, *µ_rel_*_(*b*)_) were separately evaluated. Spatial analysis was accomplished by dividing each disc into three anatomical regions: the nucleus pulposus (NP), inner annulus fibrosus (IAF), and outer annulus fibrosus (OAF). These divisions followed the contours of the disc region of interest. Within each of these regions, the values were averaged within each disc. Pearson’s correlation was used to evaluate the relationship between each pair of parameters with *n* = 3 per analysis performed. Statistical significance was defined at α = 0.05 for all tests.

## 3. Results

### 3.1. T_1_ and T_2_ Relaxation Time Mapping

**Figure 2:**
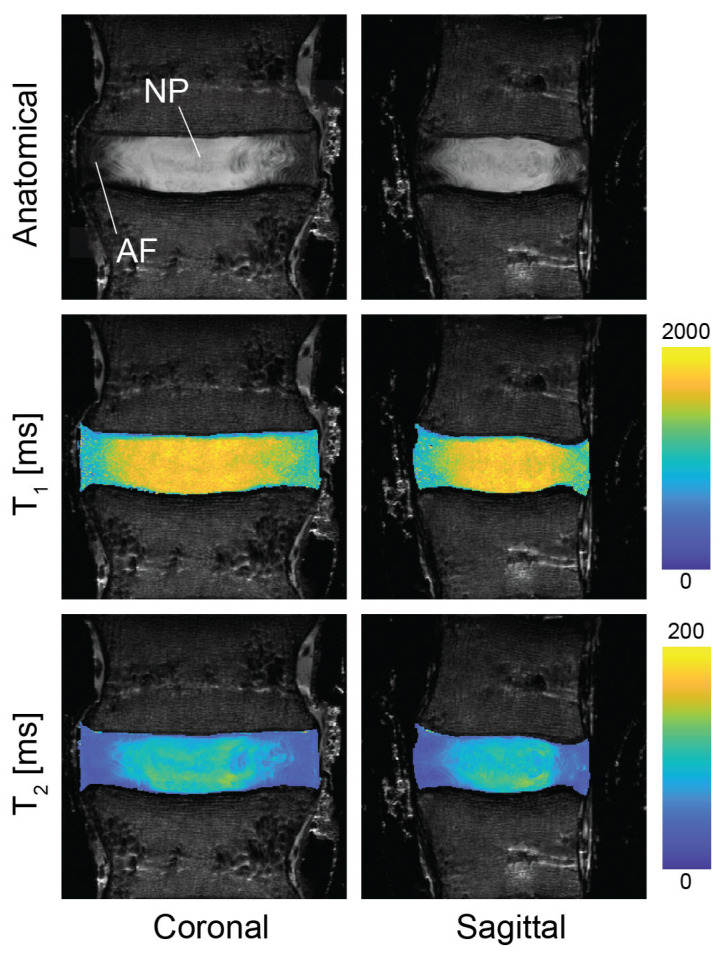
Morphological images of intervertebral discs correspond to quantitative T1/T2 relaxation time maps in coronal and sagittal imaging planes. Morphometric (proton density-weighted) images indicate a bright fluid-rich nucleus pulposus surrounded by the annulus fibrosus. Elevated values for *T*_1_ and *T*_2_ were observed in the nucleus pulposus compared to the annulus fibrosus, corresponding the fluid levels in individual tissue compartments. Relaxation time maps suggested potential imaging-based biomarkers that could also serve as surrogates for biomechanical function.

Regions of elevated *T*_1_ and *T*_2_ values were observed in the central region of the disc in both the coronal and sagittal planes (Figure 2), suggesting the location and margins of the NP and AF. The average *T*_1_ relaxation times from the coronal and sagittal planes were 1300 ± 265 and 1308 ± 270 ms, and *T*_2_ were 71 ± 29 and 71 ± 28 ms, respectively, taken across the full disc.

### 3.2. dualMRI and Strain Mapping

**Figure 3:**
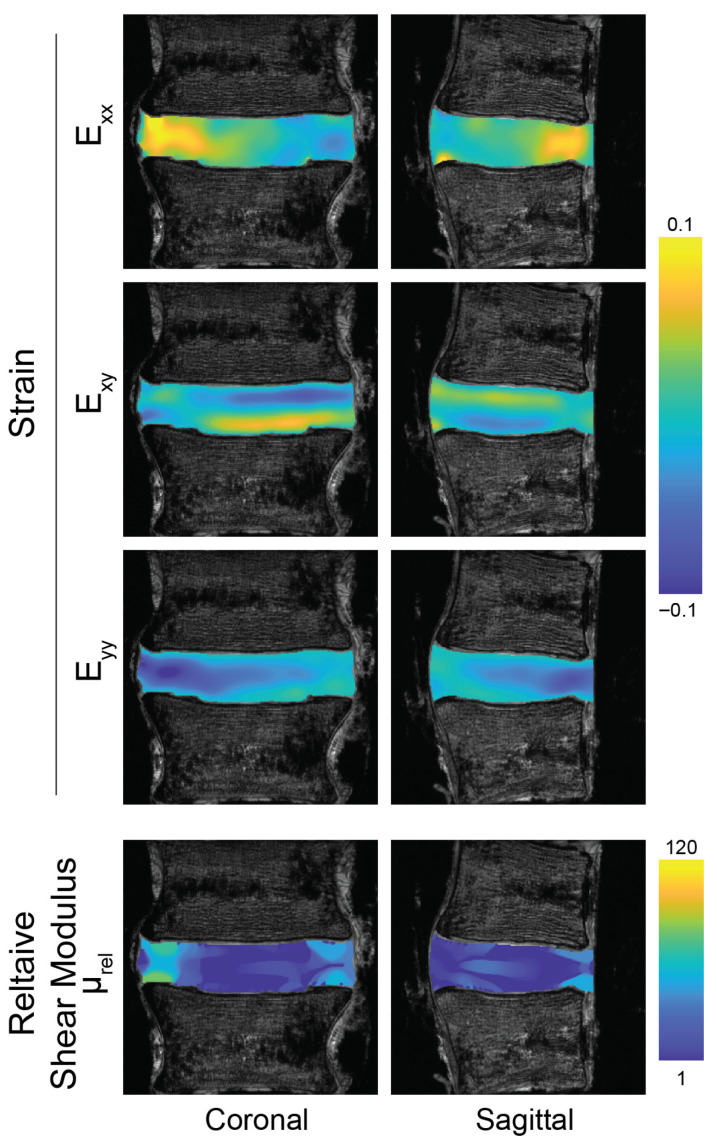
Compression of the intervertebral disc reveals complex intratissue strain patterns and regions of low stiffness in the nucleus pulposus. Green–Lagrange strains show regions of high compressive strain in the loading direction (*E_yy_*) and lateral tissue expansion (*E_xx_*), in addition to shear (*E_xy_*). Strains in coronal and sagittal image planes were calculated from displacement maps derived from displacement-encoded MRI under cyclic axial compression. Relative shear modulus was calculated separately using strain data from each image plane. Strains and moduli are shown for a representative specimen.

**Figure 4:**
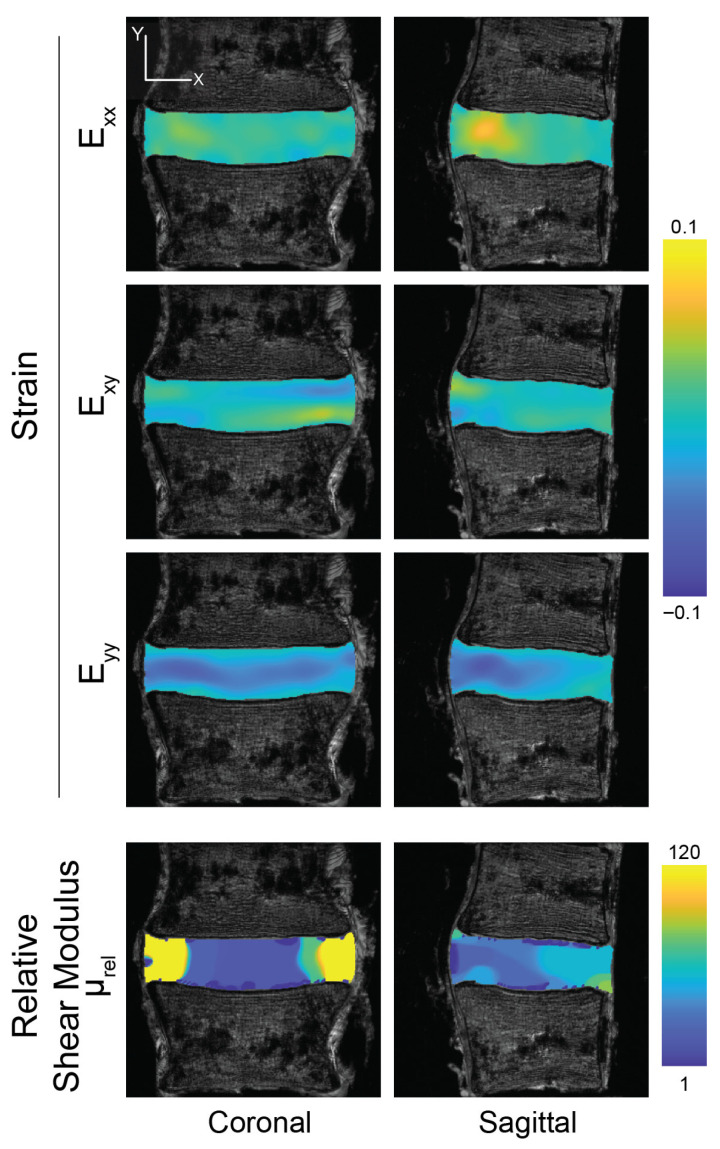
Bending of the intervertebral disc reveals region-specific intratissue strain patterns with corresponding stiffening. Intervertebral disc strain patterns with respect to the image plane (*E_xx_*, *E_xy_*, *E_yy_*), especially compressive strain in the sagittal plane show bending motion, leading to apparent softening. In the coronal plane, through which bending is applied, a more uniform compressive strain and stiffness is observed. Strains in coronal and sagittal image planes were calculated from displacement maps derived from displacement-encoded MRI under cyclic bending. Relative shear modulus was calculated separately using strain data from each image plane. Strains and moduli are shown for a representative specimen.

The strain fields, measured under compression and bending using dualMRI, were heterogeneous in both the coronal and sagittal planes (Figure 3 and Figure 4). The mean strains *E_xx_*, *E_yy_* and *E_xy_* were 0.018 ± 0.006, −0.031 ± 0.006 and 0.002 ± 0.002, respectively, taken across all discs (Table 1). The average in-plane principal strain measures (*E*_1_, *E*_2_, *γ_max_*) were 0.0166 ± 0.0075, −0.0510 ± 0.0045, and 0.0338 ± 0.0023, respectively, across all discs. Under compression, the location of the maximum *E_xx_* and *E_yy_* within the disc showed no apparent pattern in either the coronal or sagittal plane among the discs; however, under bending, the locations of the strain maxima appeared to be more consistent. In the coronal plane, the maxima were located at the midline of the disc and, in the sagittal plane, at the posterior aspect. A significant difference between compression and bending was found in the first principal strain (*p* = 0.029) and maximum shear stress (*p* = 0.013) calculated in the coronal plane but not for other mechanical parameters and not in the sagittal plane.

### 3.3. Shear Modulus

Estimates of the relative shear modulus from compression-only testing (Table 1) demonstrated stiff AFs and compliant NPs (Figure 3, lower panels). However, the discs showed an apparent stiffening in both the AFs and anterior aspects of the NP under bending (Figure 4, lower panels). The relative shear moduli estimated from strain maps taken under bending (Table 1) were significantly higher than moduli estimated with compression in the sagittal (*p* = 0.031) but not coronal (*p* = 0.133) planes.

### 3.4. Comparison of Relaxometry and Mechanical Parameters

**Figure 5:**
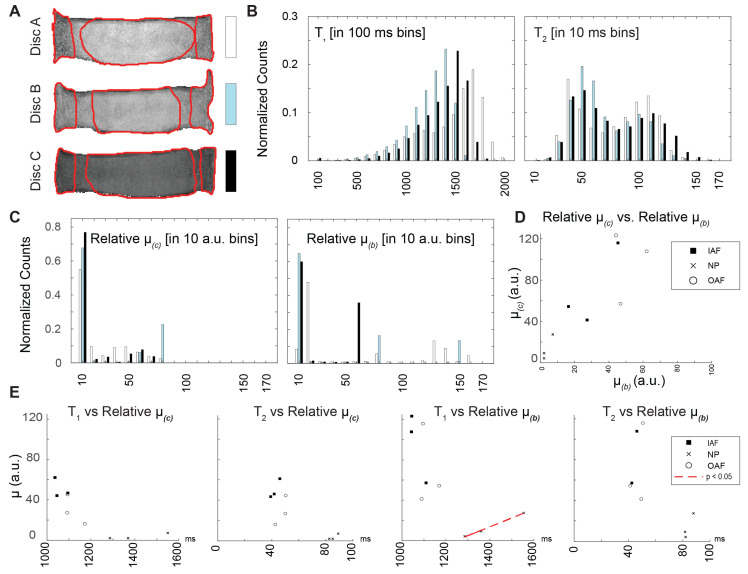
Relaxation time and relative shear moduli were correlated within the coronal slices only in the nucleus pulposus and under bending conditions. (**A**) Each disc was segmented into 3 regions for analysis of parameters in the coronal image plane: outer annulus fibrosis (OAF), inner annulus fibrosis (IAF), and nucleus pulposus (NP). Histograms (with each disc indicated with white, aqua, or black bars) of the relaxation times (*T*_1_, *T*_2_) and relative shear moduli estimated in (**B**) compression and (**C**) bending (*µ*_(*c*)_, *µ*_(*b*)_) showed no qualitative differences among discs. No correlations were found (**D**) between the relative shear moduli under bending and compression nor (**E**) among relative shear modulus and relaxation time pairs, except in the NP for *µ*_(*b*)_ and *T*_1_ (dashed line, *p* < 0.05).

**Figure 6:**
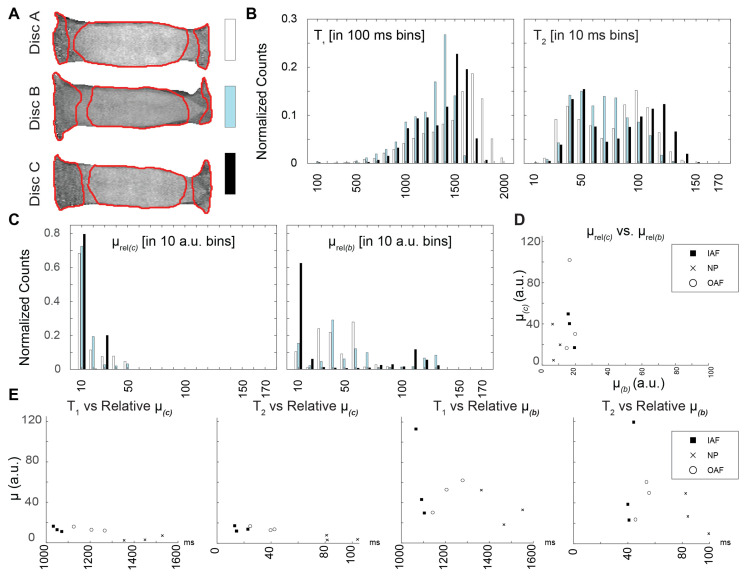
Relaxation time and relative shear moduli correlations were not observed in the sagittal images for either loading mode. (**A**) Each disc was segmented into 3 anatomic regions for analysis of parameters in the sagittal image plane: outer annulus fibrosis (OAF), inner annulus fibrosis (IAF), and nucleus pulposus (NP). Histograms (with each disc indicated with white, aqua, or black bars) of the relaxation times (*T*_1_, *T*_2_) and relative shear moduli estimated in (**B**) compression and (**C**) bending (*µ*_(*c*)_, *µ*_(*b*)_) showed no qualitative differences among discs. (**D**) Correlations between the relative shear moduli under bending and compression and (**E**) correlations between *µ*_(*c*)_ and *µ*_(*b*)_ and between relaxation times and shear moduli within each region were not statistically significant.

The relationships between the relative shear modulus and relaxation times were evaluated by region in the coronal (Figure 5) and sagittal images (Figure 6). Each disc in the coronal and sagittal planes was divided into three anatomical regions for analysis (Figure 5A and Figure 6A). The distribution of relaxation times and relative shear moduli was evaluated across all discs (Figure 5B,C and Figure 6B,C). Interestingly, the relative shear modulus estimates derived from the compression and bending experiments were better correlated—primarily in the regions that correspond to the anterior inner AF and outer NP—in the coronal plane than the sagittal plane (Figure 5D and Figure 6D). Although half of the correlations resulted in an absolute Pearson r coefficient of higher than 0.9, the only region that showed a statistically significant correlation between relaxometry and relative shear modulus was the nucleus pulposus (*T*_1_ vs. *µ*, coronal plane under bending) (Figure 5E). No other imaging plane and loading combination showed statistical significance with the relative shear modulus (Figure 5E and Figure 6E). Under compression and bending, in both the sagittal plane and coronal plane, few statistically significant correlations were found (Supplemental Figures S1 and S2).

### 3.5. Monte Carlo Simulations

Inverse analysis of a smoothed displacement map was performed, and the map of the estimated relative shear modulus was used as the reference for Monte Carlo simulations of noise-added displacements. The RMSE and precision were calculated across 100 simulations for each pixel within the region of interest (Supplemental Figure S3). Furthermore, an analysis across 100 points, randomly selected within the region of interest, from all simulations resulted in an RMSE of 1.32 (6.86%) and an average standard deviation of 0.071. Higher errors were present in the annulus region compared to the nucleus (Supplemental Figure S3). All values were unitless as they were relative shear values.

## 4. Discussion

In this study, we used displacement-encoded MRI in orthogonal (coronal and sagittal) imaging planes to evaluate the in-plane strains resulting from cyclic compression and axial bending in human lumbar intervertebral discs. To further investigate the utility of MRI relaxometry as a surrogate measure of tissue mechanical function under load, we examined correlations among relaxation times (*T*_1_, *T*_2_), relative shear modulus ($\mu_{rel}$), and in-plane principal strains (*E*_1_, *E*_2_, *γ_max_*). Because average strain measures do not capture the complexity and heterogeneity of strain patterns throughout the tissue, spatial mapping of mechanical behavior is needed. Although fully three-dimensional dualMRI would require impracticably long imaging times, doubling the acquisition time to measure strains in orthogonal directions may provide valuable insight into how the tissue volume responds to different loading conditions. The goals of this study were (1) to evaluate MRI relaxation times and dualMRI-derived mechanical parameters such as strain and estimated modulus in orthogonal anatomic planes under two common disc-loading modes and (2) to determine whether MRI relaxation times and mechanical parameters can serve as mutual surrogates for characterizing disc heterogeneity.

The mechanical function of the disc is closely associated with the structure and content of the disc ECM [44]. Although relaxometry offers a straightforward measurement of disc composition, it remains unclear whether the relaxation time can act as a sole indicator of disc material properties or mechanical behavior. An indirect link is plausible, because qMRI reflects biochemical content and structure, which influence the tissue’s mechanical behavior. Thus, qMRI may complement deformation patterns measured under load, as previously demonstrated in articular cartilage using qMRI and dualMRI [44]. However, prior work comparing principal strains with *T*_1_*_ρ_* and *T*_2_ relaxation times in vivo found no statistically significant correlations [45], suggesting that the direct measurement of disc mechanical behavior under load may provide a more comprehensive assessment of disc health, including potential pain-generating mechanisms, than structural or compositional biomarkers alone.

Previous studies have estimated disc deformation in vivo by measuring nominal disc height changes using MRI [46], video fluoroscopy [47], ultrasound [47], and dynamic radiographic imaging [48]. These methods rely on nominal measures (e.g., distances between endplate surfaces) but do not capture the internal mechanical behavior needed to detect the focal heterogeneous changes associated with degeneration [49]. Deformable image registration techniques (e.g., warp field, digital image correlation) have been applied to measure the strain in the AF by tracking intrinsic textural features in morphological images [50]. In contrast, the phase-encoded data in dualMRI allow measurement of tissue displacements with high precision and resolution, independent of image texture [26]—an advantage for disc imaging because the NP often lacks trackable structural features.

We measured the in-plane displacement in two orthogonal planes (coronal and sagittal, through the disc midpoint) using displacement-encoded MRI and calculated the resulting full-field strains, whose magnitudes were consistent with the previously reported values [21]. We observed similar displacement magnitudes and patterns in the loading (y) direction under compression within the sagittal and coronal planes. As expected, the displacement and strain patterns differed between loading modes in the sagittal plane, where high-strain regions shifted anteriorly during bending. Unlike the idealized deformations of a prismatic homogeneous specimen, transverse (x) displacements under compression were unevenly distributed across the specimen width in both planes, indicating that complex geometry and heterogeneous material properties drive mechanical behavior. Because the discs were at least Pfirrmann grade 2, these deformation patterns likely reflect early to moderate degeneration. These results highlight the complementary value of measuring full-field strains in orthogonal anatomic planes, under different loading conditions, to better characterize the complex mechanics of heterogeneous tissues such as the disc. Likewise, image-based computational models incorporating complex geometries and regional variations in material properties will likely better replicate in situ and in vivo deformation patterns.

Regional correlation analysis revealed limited evidence of relationships between mechanical behaviors measured with dualMRI and relaxation times *T*_1_ and *T*_2_. Among correlations with relative shear modulus, only one region showed statistically significance between *T*_1_ and *µ_rel_*_(*b*)_ (NP in the coronal plane, *p* = 0.040). Previous studies have shown associations between relaxometry and deformation under compressive loading [23], as well as bending stiffness [15], and that these values remain stable during moderate cyclic loading [35]. The absence of correlations between *T*_2_ and the relative shear modulus contrasts with earlier findings in the NP [28], wherein MRE was applied to 16 cadaveric discs; however, that study examined only the NP. Other studies have reported inconsistent correlations between *T*_1*ρ*_ and radial or axial AF strains [23]. Taken together, the lack of significant correlations across multiple disc regions suggests that MRI relaxometry outputs may not currently serve as direct proxies for localized mechanical parameters or vice versa. The limited correspondence between the estimated material properties and the relaxometry could also be due to the boundary conditions, inputs upon which the inverse model relies. Displacements were measured as the difference between the loaded and unloaded portion of the cyclic loading cycle; thus, the magnitudes were dependent on the loading modes and the fixed connection to the loading device of the inferior vertebral body. However, *T*_1_ and *T*_2_ distributions would not change with the loading mode, while displacements, and thus the inferred moduli, would change spatially with loading. This discrepancy also implies that relaxometry and mechanical parameters should be assessed independently, consistent with previous cervical spine research showing weak strain–relaxation correlations [26]. These ambiguous relationships may indicate a disconnect between material properties and mechanical behavior in in vitro studies such as this and others [51], where discs were removed from their physiological environment and loaded along simplified degrees of freedom. A separate study examining discs with higher Pfirrmann degeneration grades (III to V) found significant correlations between proteoglycan and collagen content and mechanical properties, such as storage and loss modulus [52]. Together with our findings, this suggests that some mechanical properties may correlate better with biochemical content than others. These results further underscore the need to measure or model mechanics and composition within the native mechanical environment of the intact spine and under physiologic loading conditions that reflect daily activities contributing to back pain.

We also estimated the relative shear modulus using an iterative inverse approach that minimizes differences between measured and computed displacement fields. Substantial variance in the estimated relative shear modulus across samples was observed within the same regions. For example, in the central region, the average relative shear modulus was 27.45 ± 23.15 across three samples, as estimated in the sagittal plane under bending. Several factors may contribute to this high variability, including inter-donor differences and image noise, which increases the error in displacement maps and thus in the inverse model. Modulus estimates derived from compression experiments indicated some AF regions appearing ~50 times stiffer than the NP, while estimates made from bending experiments reached relative values up to 120. Interestingly, the high relative values under bending occurred only in the coronal, but not sagittal, plane. In the bending mode, the coronal slice showed higher shear strains in the AF than NP, potentially overweighting the estimated modulus in that region. Furthermore, prior MR elastography reported the AF shear modulus to be approximately 10 times higher than the NP [28]. Although the AF-NP modulus ratio varies with degeneration [53], the magnitude of the estimated values here was unexpectedly large. This discrepancy could be due to the differences in response to load–displacement frequency in a viscoelastic tissue. The previous MR elastography study used displacement oscillations measured under 1250 Hz [28], while the discs in this study were loaded cyclically at 0.2 Hz, suggesting that the frequency of the loading could have an effect on the predicted moduli, as was shown under torsional loading and MR elastography [54]. Monte Carlo simulations were also used to evaluate the model error, using the relative shear modulus estimated using coronal plane deformations under compression. The 100 simulations yielded a normalized RSME of 0.0686 (6.86%) and a mean standard deviation of 0.071. A previous study on articular cartilage using a similar approach found bias and precision values of 0.092 and 0.062, respectively, on a 0–1 relative shear modulus scale [50], comparable to our normalized results.

When comparing the relative shear moduli derived from compression versus bending experiments, correlations were observed in the coronal, but not the sagittal, plane. Because sagittal strains differed substantially between loading modes, the absence of correlations in this plane is not surprising. The differing load boundary conditions between loading modes inherently alter the model inputs. In the coronal plane, where in-plane strains were expected to be more similar between loading modes, correlations emerged in only some regions. Overall, the limited agreement in relative shear moduli between loading regimes and between relative shear moduli and relaxation times highlights the limitations of treating loading conditions as independent. Continued development of inverse methods may improve the accuracy by constraining multiple deformation datasets, acquired in orthogonal planes or throughout the full tissue volume, to a unified nonhomogeneous model.

Several limitations to our MRI-informed inverse approach may explain the wide range of modulus estimates and suggest directions for future refinement. As noted above, differences in modulus prediction between this study and prior MR elastography studies could be due to the assumptions made within our model. Both the NP and AF were assumed to be isotropic and linearly elastic, which does not consider the fiber direction of the AF. Future work could potentially incorporate diffusion tensor imaging or other MRI techniques that are sensitive to collagen fiber alignment [55], enabling the inclusion of fiber orientation in the AF in this MRI-based approach. Furthermore, the constitutive model assumes tissue incompressibility based on high water content. However, reported Poisson’s ratios range from ~0.12 [56] to 0.62 ± 0.15 [57] for NP and vary even more widely in AF, depending on the loading direction and other factors [58]. Thus, future work could also benefit from the incorporation of water content [59] and diffusivity [60] using MRI-based measures. Additionally, although this model previously succeeded in recovering shear modulus distributions under uniform compression [42], the strain patterns in this study were nonuniform, even during compression, which likely amplified the errors. Nonuniform displacement fields during bending may further explain the larger modulus estimates in this loading mode, especially the higher estimations for the AF in the coronal plane noted above. The inverse method also assumed plane-strain conditions for each imaged slice. Moreover, the 9.4 T magnetic field used here exceeds the strength of most clinical scanners (typically ≤3 T). Prior work has shown comparable displacement measurements between 9.4 T and 3 T [35], suggesting the modulus estimates should also be comparable. Another limitation is the small sample size of three cadaveric discs, with minimal medical background available. Lastly, although displacement fields were smoothed, local variations from image noise may still affect modulus estimation. Given these constraints in this initial application of the iterative inverse approach to displacement-encoded MRI, further methodological advancements will be essential for achieving higher accuracy in elastography at physiologic loading frequencies.

## 5. Conclusions

In summary, we applied noninvasive displacement-encoded MRI to measure disc deformation under cyclic axial compression and anterior bending. Full-field deformations, shear modulus, and relaxation times (*T*_1_**,** *T*_2_) were quantified in the coronal and sagittal planes. This work demonstrated that acquiring displacement-encoded MRI in orthogonal planes can yield richer deformation information without the time burden of full-volume imaging. Inverse modeling enabled the estimation of the relative shear modulus and an exploration of its relationship with relaxation times, although the correlations were inconsistent and dependent on the loading mode. Thus, image-based elastography and relaxometry appear to provide complementary, but not interchangeable, measures of disc structure and function, with potential applications in longitudinal disc assessment. Future development of reliable methods for measuring in vivo deformation during physiologically relevant motions (e.g., bending, twisting) and estimating disc material properties will be essential for understanding how mechanical behavior relates to the onset and progression of low back pain in patient-specific analyses.

## Figures and Tables

**Table 1:** Average mechanical parameters across all discs. In-plane strain calculated from displacement-encoded MRI and relative shear moduli ($\mu_{rel}$) were averaged over the entire disc regions of interest in the indicated image planes, such that stiffer tissues have a higher $\mu_{rel}$. Strains were calculated with respect to the image plane (*E_xx_*, *E_yy_*, *E_xy_*) as well as in in-plane principal directions (*E*_1_, *E*_2_) and maximum shear strain (*γ_max_*). Data represent mean ± standard deviation (*n* = 3 biological replicates). Significant differences in a mechanical parameter estimated from compression vs. estimated from bending experiments are indicated in bolded *p* values.

Mechanical Parameter	Image Plane	Compression	Bending	Paired *t* Test (*p*)
*E_xx_*	Coronal	0.017 ± 0.003	0.010 ± 0.001	0.061
	Sagittal	0.023 ± 0.002	0.023 ± 0.004	0.835
*E_yy_*	Coronal	−0.035 ± 0.005	−0.034 ± 0.008	0.924
	Sagittal	−0.026 ± 0.005	−0.0311 ± 0.006	0.189
*E_xy_*	Coronal	0.001 ± 0.002	0.001 ± 0.002	0.505
	Sagittal	0.003 ± 0.001	0.002 ± 0.001	**0.039**
*E*_1_	Coronal	0.017 ± 0.007	−0.001 ± 0.005	**0.029**
	Sagittal	0.029 ± 0.005	0.022 ± 0.004	0.127
*E*_2_	Coronal	−0.051 ± 0.004	−0.048 ± 0.005	0.700
	Sagittal	−0.035 ± 0.010	−0.037 ± 0.008	0.814
*γ_max_*	Coronal	0.034 ± 0.002	0.024 ± 0.003	**0.013**
	Sagittal	0.031 ± 0.004	0.030 ± 0.006	0.923
$\mu_{rel}$	Coronal	18.3 ± 5.8	38.9 ± 16.8	0.133
	Sagittal	8.4 ± 1.6	50.4 ± 13.0	**0.031**

## Data Availability

The original data presented in this study are openly available in the Purdue University Research Repository at https://doi.org/10.4231/49GD-ES15.

## Supplementary Materials

The following supporting information can be downloaded at https://www.mdpi.com/article/10.3390/bioengineering13040437/s1, Figure S1: Correlations by region within the disc between relaxation times and calculated strains from dualMRI in the coronal plane within each anatomical region; Figure S2: Correlations by region within the disc between relaxation times and calculated strains from dualMRI in the sagittal plane within each anatomical region; Figure S3: Monte Carlo results in a single disc under compression in the coronal plane.
