# Supplementary material for: Intervertebral Disc Elastography to Relate Shear Modulus and Relaxometry in Compression and Bending
Source: Bioengineering (Basel). 2026 Apr 8;13(4):437. doi: 10.3390/bioengineering13040437 (PMC13113507; doi:10.3390/bioengineering13040437)
Supplement: Supplementary file 1 [file bioengineering-13-00437-s001.zip › bioengineering-4203124-supplementary.pdf]

SUPPLEMENTAL DATA

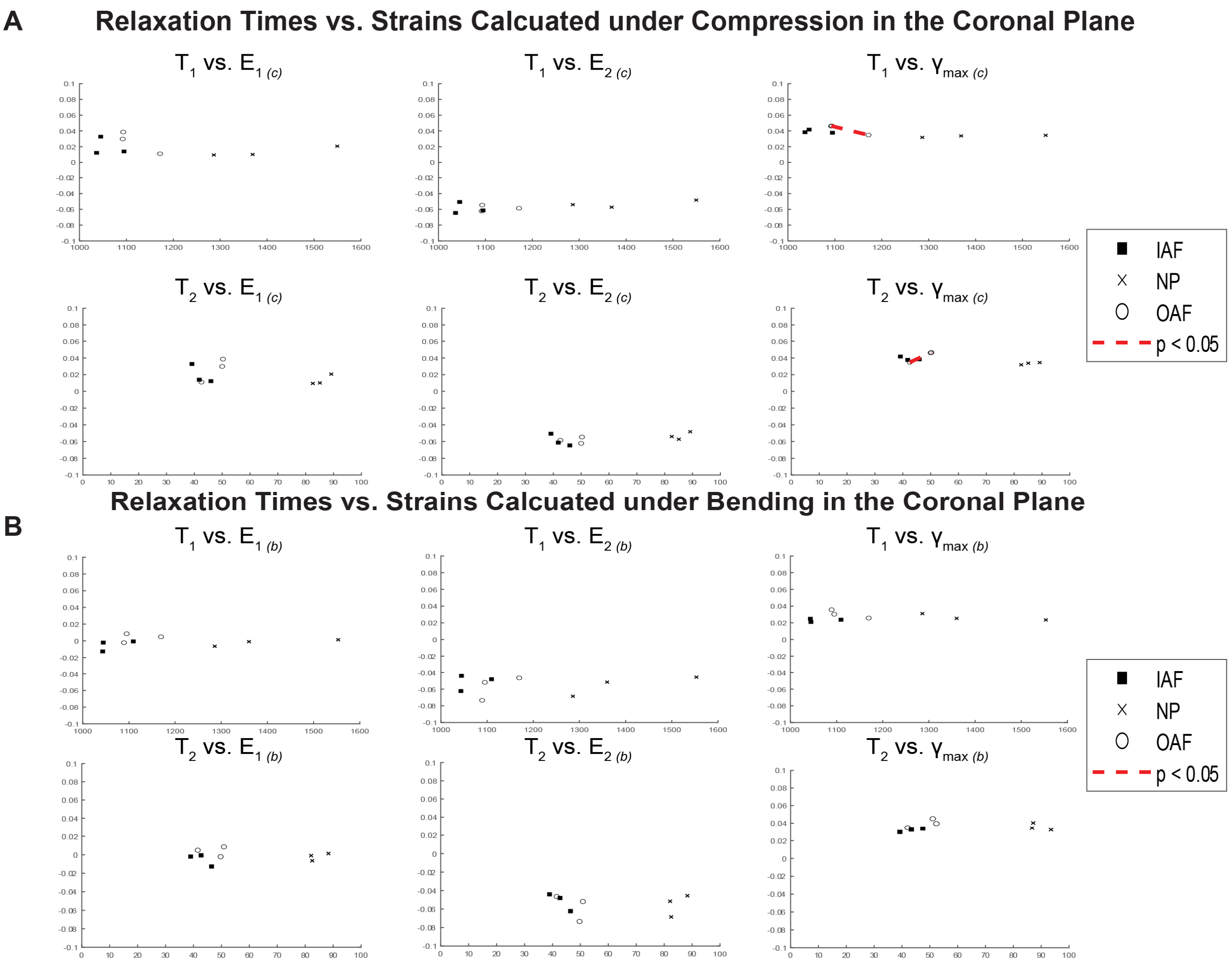

Supplemental Figure S1. Correlations by region within the disc between relaxation times and calculated strains from dualMRI in the coronal plane within each anatomical region (IAF = inner annulus fibrosus, NP = nucleus pulposus, OAF = outer annulus fibrosus).

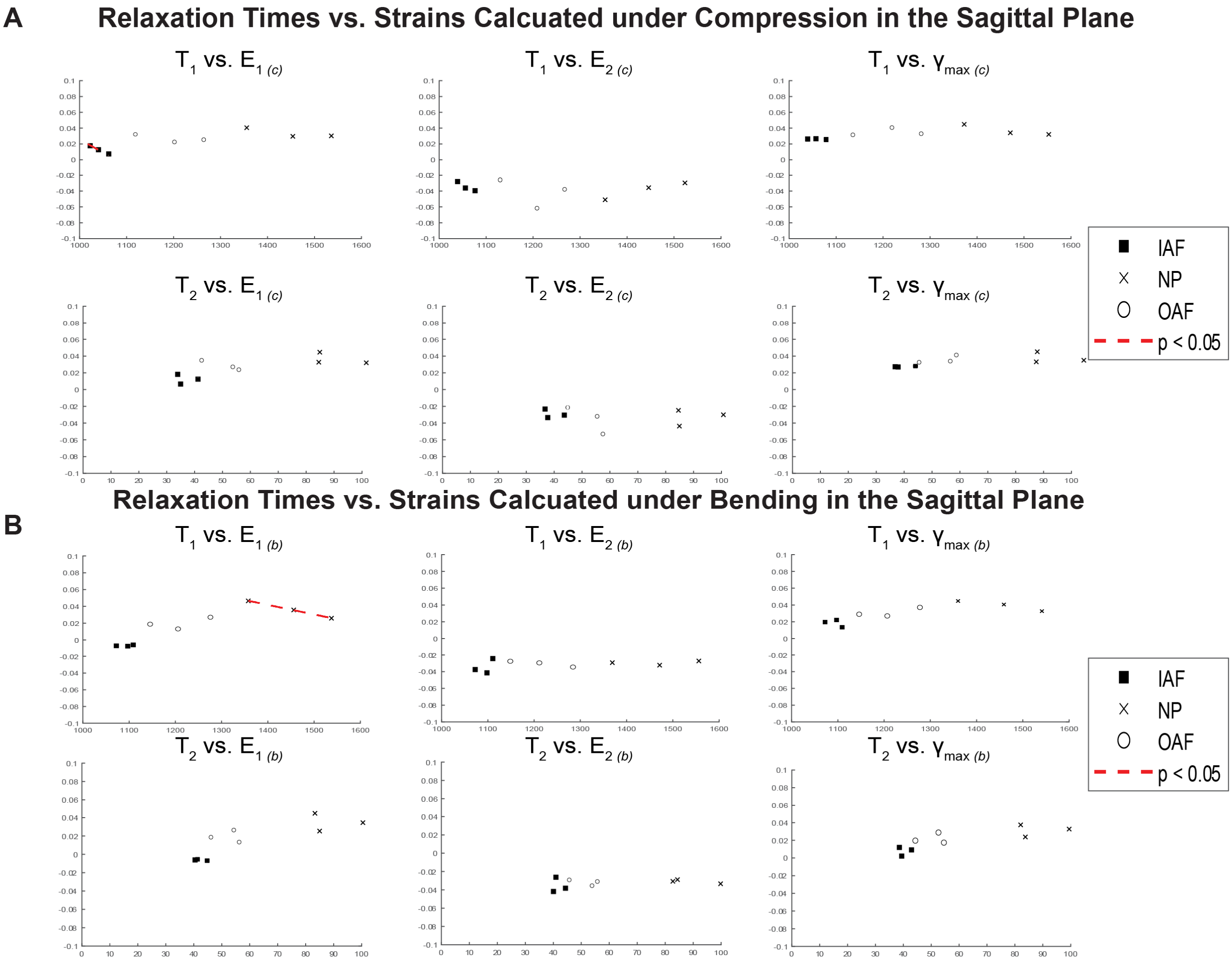

Supplemental Figure S2. Correlations by region within the disc between relaxation times and calculated strains from dualMRI in the sagittal plane within each anatomical region (IAF = inner annulus fibrosus, NP = nucleus pulposus, OAF = outer annulus fibrosus).

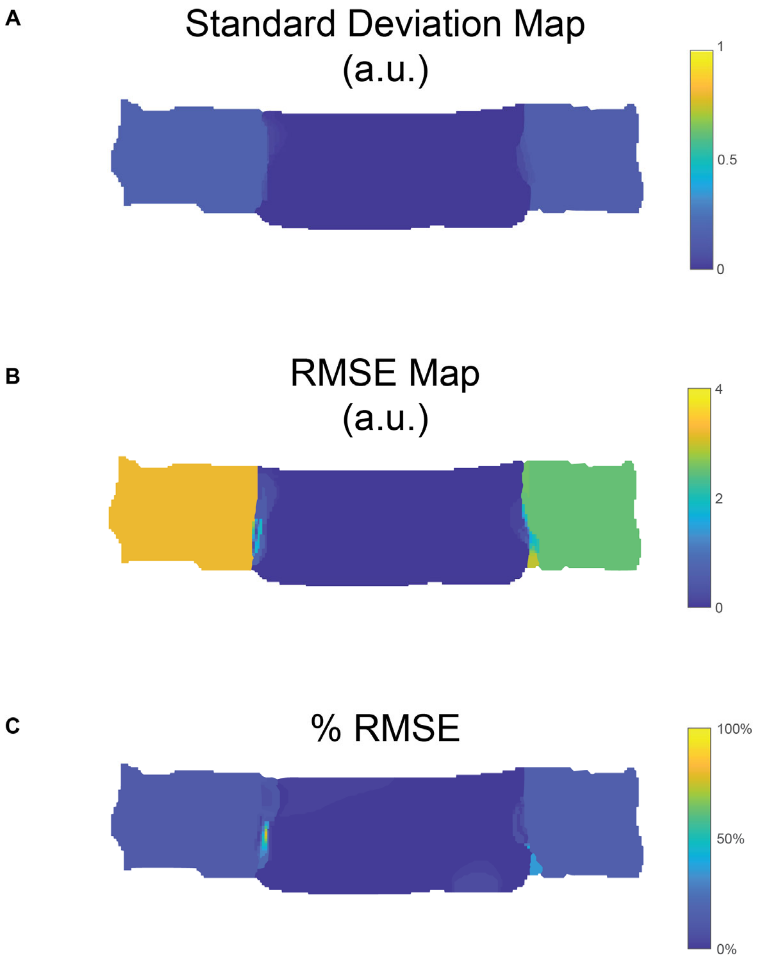

Supplemental Figure S3. Monte Carlo results in a single disc under compression in the coronal plane. Maps of (A) standard deviation, (B) absolute RMSE, (C) RMSE as a percent of relative shear modulus at each pixel, all of which were evaluated across the 100 simulations.
